# Impact of Initial Electron Localization on Electron Solvation Dynamics in Liquid Water

**DOI:** 10.1021/acs.jpclett.6c01216

**Published:** 2026-06-26

**Authors:** Nathaniel Okpara, Mathilde Goullieux, Ludger Inhester, Robin Santra

**Affiliations:** † Center for Free-Electron Laser Science CFEL, Deutsches Elektronen-Synchrotron DESY, Notkestr. 85, 22607 Hamburg, Germany; ‡ Department of Physics, University of Hamburg, Notkestr. 9-11, 22607 Hamburg, Germany; ¶ The Hamburg Centre for Ultrafast Imaging, Luruper Chaussee 149, 22761 Hamburg, Germany

## Abstract

The solvation mechanism of an excess electron in liquid water has been extensively investigated, yet significant controversies persist regarding the time scales, dynamics, and precise localization processes of this quantum species. Here, we employ ab initio molecular dynamics simulations to elucidate the impact of the degree of initial localization of an excess electron on the solvation process in liquid water. We demonstrate that the degree of initial delocalization of the excess electron critically governs its solvation dynamics. Furthermore, we reveal the role of excited-state dynamics in modulating solvation time scales and discuss the heating of the water structure due to the energy released via internal conversion to the electronic ground state. Our results reveal that the way how the electron is injected critically determines its solvation dynamics.

## Graphical abstract

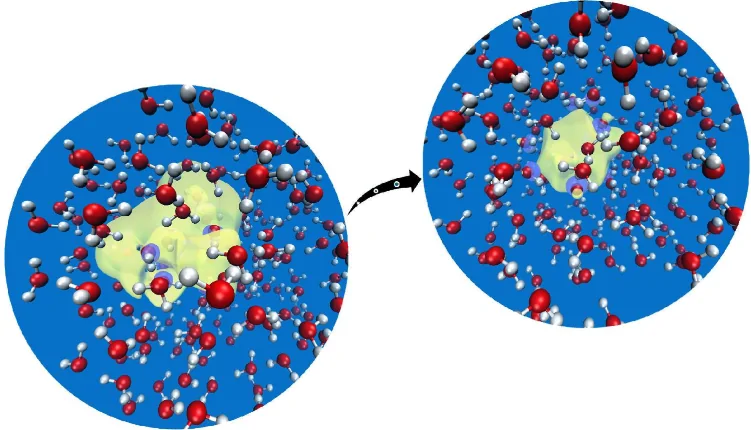

The hydrated electron, first characterized spectroscopically by Hart and Boag,^1^ is a pivotal transient species in radiation chemistry. Its high reactivity underpins critical processes in radiobiology, particularly by influencing DNA damage pathways,^2,3^ governing radical reaction dynamics,^4^ driving degradation of critical pollutants,^5^ and mediating corrosion in nuclear reactor systems.^4^ This significant chemical impact has, for decades, driven efforts to understand its fundamental physical nature.^2,6,7^ With respect to its microscopic structure, existing studies largely agree that the electron is trapped in a potential well inside an excluded volume in the structure of liquid water. The region is confined by 4–6 water molecules, pointing their O–H bond toward the center of the excess electron density.^7^ Even though contradicting models were discussed,^8^ the picture of an electron inside such a cavity is now generally accepted.^2,7,9^ How such a cavity structure emerges is, however, not fully resolved. When an electron is injected in bulk water by photoionization, the electron is usually assumed to be initially largely delocalized. Immediately, its excess energy will dissipate into the surrounding water molecules until the interaction with water molecules ultimately triggers the formation of a cavity structure accompanied by the contraction of the excess-electron density.

Time-resolved photoabsorption,^10–18^ photoelectron spectroscopy,^19,20^ and time-resolved X-ray absorption spectroscopy^9^ experiments indicate a time scale of several 100 fs for this solvation process, sometimes augmented with a secondary long-time component of about 1 to 2 ps.^19,20^ Interpretations diverge on whether the solvation can be characterized as a sequence of distinct steps^12^ and how appropriate precursor states should be characterized.^15,21,22^ The initial phase of the solvation process is sometimes characterized as a pure trap-seeking process.^23–26^ According to this concept, the initial excess-electron density is so diffuse that it has negligible impact on the structure of the water molecules. The excess electron contracts at a preexisting trap in the water structure, a favorable site that spontaneously emerges from the fluctuations of the water structure. Regardless of the extent to which the role of the excess electron in this process can be considered entirely passive, its contraction is accompanied by a decrease in its binding energy as well as a change in the associated absorption spectrum.^7^ In the most recent works, this first step of the electron-solvation dynamics has been associated with a time scale of about 200 to 300 fs.^9,22^ The trap-seeking step is followed by further reshaping of the local structure of the water molecules.^9^ This active trap-digging process does not only involve the nearby water molecules but, on longer time scales, also the equilibration of the water structure farther away. Even though a number of theoretical models for the equilibrium state exist,^6^ simulations focusing on the solvation after injection into bulk water are scarce.

Rossky and Schnitker pioneered the theoretical description of the electron-solvation process^27^ using a one-electron-quantum-mechanics/molecular-mechanics (QM/MM)-embedding scheme with pseudopotentials for the interaction between water and the excess electron. Surface-hopping simulations with this model indicated that the excess-electron–density contraction occasionally precedes the relaxation to the electronic ground state. This suggests that the solvation process involves a transient excited electronic state of the electron inside the cavity.^28–30^ The time scales provided in these computational studies were lower than those observed in experiments (about 25 fs). More recent works based on adiabatic molecular dynamics (MD) simulations employing density-functional theory (DFT)^18,21^ and time-domain-DFT-based nonadiabatic MD simulations^22^ revealed time scales for the contraction of the excess-electron density of several 100 fs up to 1 ps, with contrasting pictures on possible transient precursor states. Sopena Moros et al. employed path-integral MD simulations based on a machine-learning-model force field trained on the anionic ground state computed with unrestricted second-order Møller–Plesset perturbation theory (MP2).^9,31^ From an ensemble of trajectories, times for the contraction of the excess-electron density between 500 fs and 2 ps were obtained, indicating a large sensitivity to the initial temperature.

The somewhat incoherent picture drawn by these works, motivates the exploration of alternative modeling schemes and identification of the relevant mechanisms. We note that addressing electron solvation via ab initio MD simulations is computationally demanding, because of the large size of the system that is inherently involved in a transition from a largely delocalized to a rather localized species. Generally, there exist two different computational strategies to model the bulk of liquid water for such a problem. One can mimic bulk properties either by imposing periodic boundary conditions (PBC) or by embedding the electronic structure calculation in a larger force field MD environment using QM/MM embedding. The use of PBC might introduce artifacts due to spurious interactions between periodic images and lack of heat dissipation. Furthermore, the degree of initial electron delocalization is essentially limited to the chosen box size. On the other hand, QM/MM embedding simulations also artificially constrain the spatial region that the excess electron can occupy and, in addition, introduce artificial QM/MM boundary effects. On the electronic-structure model side, DFT methods inherently involve the choice of functional, which turned out to considerably impact the structure of the solvated electron.^32,33^ It has been shown that the Hartree–Fock method is able to yield stable cavity structures similar to those obtained by incorporating dynamical correlation effects via MP2 from refs ( ^9^,^31^ ).^34,35^

Moreover, due to the statistical nature of the process, it is required to run multiple trajectories sampling the initial conditions that represent the fluctuations in bulk water at normal conditions. In light of these challenges, we here investigate the electron solvation as captured by Hartree–Fock-based QM/MM-embedding simulations. We specifically address two key questions that in the earlier simulation works have not been addressed: How do the solvation dynamics depend on the degree of delocalization? How are the solvation dynamics modified when the electron is initially injected in an excited electronic state?

To address these questions, we present molecular-dynamics-simulation results based on two different simulation frameworks. We performed adiabatic MD simulations based on unrestricted Hartree–Fock (UHF) electronic-structure calculations for the anionic ground state. Moreover, in order to address initially excited states, we also performed nonadiabatic surface-hopping simulations based on Koopmans’ theorem for electron addition. The associated states are constructed by adding an electron to the different virtual orbitals (i.e., the electron may or may not be in an excited state) of the neutral Hartree–Fock ground state. Both frameworks employ QM/MM embedding, whereby the electronic structure is propagated for a cluster of 12 water molecules and the surrounding water molecules are treated using a force field to model bulk properties (see further details in Computational Details).

Employing the UHF framework, the solvation dynamics were initiated by switching from the neutral ground state to the anionic ground state for an ensemble of MD trajectories. Figure 1 summarizes the time evolution of the spin density upon injection of the excess electron for different basis sets (6-31+G,^36,37^ 6-31++G,^38^ cc-pVDZ,^39^ and aug-cc-pVDZ^40^). In the top row, ensemble percentiles for the radius of gyration of the spin density *R*_*G*_ are shown as a function of time. This quantity directly reflects the degree of localization of the excess electron. As can be seen, the spin density of the excess electron for all basis sets exhibits rapid contraction of the electron’s radius of gyration *R*_*G*_ from initially delocalized states with *R*_*G*_ > 3.0 Å to more localized states. The spatial extent of these final states is characterized by an *R*_*G*_ of about 2.4 Å for the more compact basis sets (6-31+G, cc-pVDZ) and 3.0 Å for the basis sets incorporating additional diffuse basis functions (6-31++G, aug-cc-pVDZ). For isolated solvated-electron-cavity structures, a similar basis-set dependence is discussed in ref (^34^). A more detailed picture of the radial distribution function of the spin density and its center is given in Sections S1 and S5 in the Supporting Information (SI). The contraction of the spin density indicates that the electron becomes solvated—it localizes into a finite spatial region, implying that the water molecules around the excess electron undergo reorientation and build up a cavity structure (see Section S4 in the SI). While the basis set influences the final value for *R*_*G*_, the fundamental physics of electron hydration remains robust across the different basis sets all giving rise to solvated structures.

**Figure 1:**
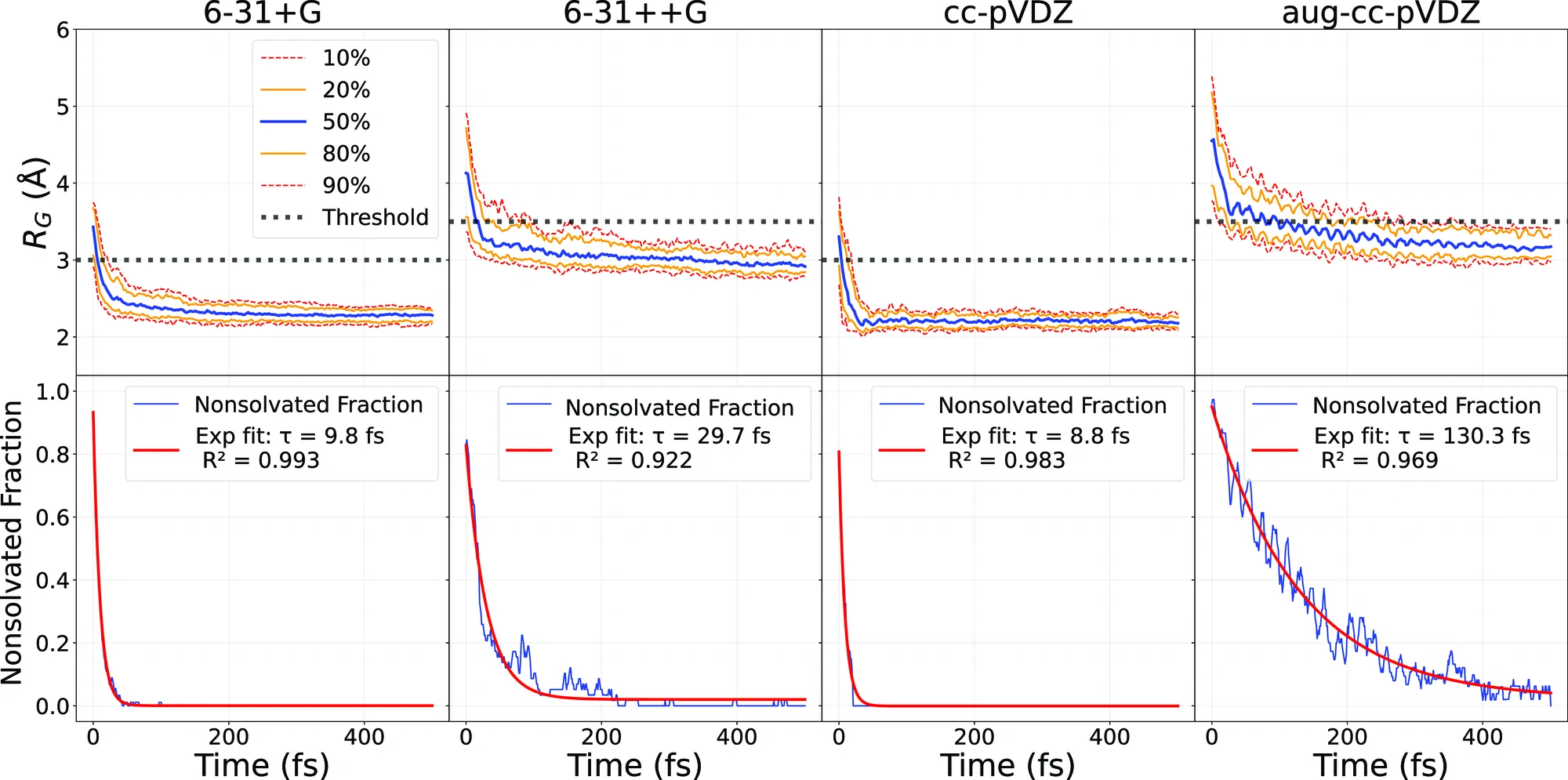
Contraction of the spin density in the ground state. Top row: Evolution of the radius of gyration of the spin density (*R*_*G*_). The lines show ensemble percentiles (10%, 20%, 50%, 80%, 90%). Dashed lines indicate cavity formation thresholds. Bottom row: Fraction of trajectories with nonsolvated electrons.

Each individual trajectory was inspected and the solvation state was determined using a threshold of 3.0 Å for the 6-31+G and cc-pVDZ; and 3.5 Å for the 6-31++G and aug-cc-pVDZ basis set (see Section S1 in the SI for a discussion on these thresholds). The evolution of the fraction of trajectories not yet solvated is shown in the bottom row of Figure 1. The red lines show exponential fits to the data, quantifying solvation time constants τ for each basis set. The obtained time scales diverge significantly: the more compact basis sets (6-31+G, cc-pVDZ) achieve excess-electron density contraction within <30 fs, whereas the diffuse-augmented basis sets (6-31++G, aug-cc-pVDZ) require nearly ∼30 to 100 fs.

Electronic-structure calculations on discrete states may, in principle, be converged by increasing the basis-set size. Such a convergence, however, cannot be expected when intrinsically delocalized states in a continuous electron band are targeted. This is the case for the electron injected in the conduction band of liquid water. The description of the excess electron in the simulation is therefore fundamentally constrained by the size of the QM region and the basis set. At the same time, in a time-resolved measurement, it is fundamentally impossible in the pump step to populate only a single eigenstate in the electronic continuum. The resulting superposition of eigenstates generally leads to a more spatially confined excess-electron density than would be the case for the individual eigenstates. Here, we make use of the constraints imposed by the limited basis set and the finite QM region in order to impose a spatially confined initial excess-electron density. Particularly, in this work, the size of the basis set serves as a tool that allows us to control the degree of initial localization of the excess electron. The presence of the additional diffuse functions in the 6-31++G and aug-cc-pVDZ basis sets gives rise to a larger degree of delocalization of the initially unsolvated excess electron as compared to the delocalization obtained using the 6-31+G and cc-pVDZ basis sets. Larger delocalization gives rise to a lower local excess-electron density that leads to weaker interaction with individual water molecules. Thus, larger delocalization reduces the ability of the excess electron to induce the molecular reorientation necessary for cavity formation. Conversely, we attribute the faster dynamics for the more compact basis sets to the fact that the excess electron already starts in a considerably more localized state.

To further quantify this effect, Figure 2 shows a scatter plot, exhibiting the initial radius of gyration vs solvation time for each trajectory. As can be seen, there is a clear trend that the solvation time increases with higher initial radius of gyration. This trend is also observed for different sizes of the QM region, as shown in Section S6 in the SI. The comparison validates the hypothesis that increased initial smearing out of the electron over a spatial region, which gives rise to a more conduction-band-like character,^14^ retards solvation dynamics. Simulations employing the more compact basis sets necessarily start with a more localized electron. Consequently, they imply a higher local excess-electron density that leads to stronger interactions with the water structure and thus to more rapid cavity formation. Conversely, simulations with diffuse basis sets, that capture greater initial delocalization and, therefore, a lower local excess-electron density, yield a longer solvation time. Because this trend is consistent across the different basis sets, we argue that the major effect resulting from the variation of basis sets is the spatial extent of the initially injected electron.

**Figure 2:**
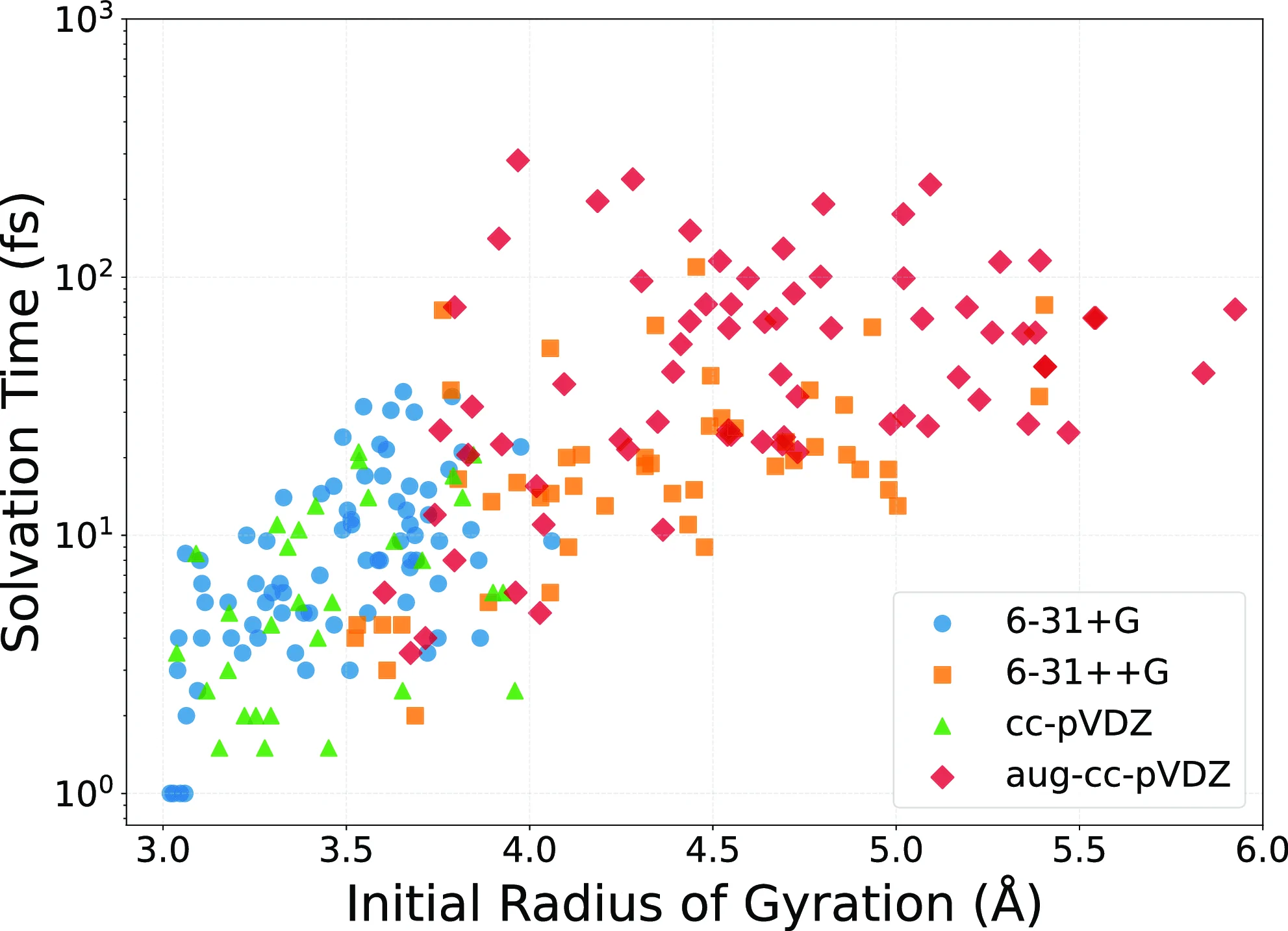
Scatter plot showing solvation time vs initial radius of gyration of the spin density for each trajectory. The solvation time is defined by the time the radius of gyration is below a specific threshold (see the text). Note the logarithmic scale used for the vertical axis.

These results can be contrasted with the work of Sopena et al., who employed PBC. In that case, the excess electron was initially spread over the whole simulation box (edge length 11.3 Å) and much longer excess-electron contraction times (0.5 to 2.0 ps^9^) were found. This is consistent, in view of the arguments we have presented, with a suppressed ability of a strongly delocalized excess electron to drive cavity formation.

We conclude that the degree of initial spatial localization of the excess electron, which in our case is governed by the diffuseness of the basis set, is one of the primary determinants of its solvation dynamics. This may explain why the solvation rate is contingent on the initial preparation of the electron.^17,18,34,41^

So far, we have addressed how the excess-electron density contracts when it is injected into the lowest electronic state. We now consider how its density contracts when the excess electron is injected into some electronically excited state. To that end, we employed the basis set 6-31+G and simulated the trajectories with the Koopmans’ theorem scheme. In contrast to the earlier simulations, where the trajectories were propagated only on the electronic ground state, here, we modeled the dynamics involving several potential energy surfaces using the fewest-switches-surface hopping (FSSH) method.^42,43^ To illustrate the resulting dynamics, Figure 3(A) shows the electronic potential energies of selected excited states as a function of time for a representative trajectory. The potential energy of the active electronic state is highlighted by the red line, revealing how the active state jumps from state D5 to D4, then D3, D2, D1, and finally down to the anionic ground state D0. This relaxation cascade involves an overall decrease of potential energy of about 4 eV. Employing the same initial conditions as before, we computed ensembles of several trajectories with the excess electron injected into different virtual orbitals (see Computational Details). In the following, we categorize the trajectories based on the excess electron’s initial molecular orbital energy, i.e., the injection energy of the excess electron ϵ, and use the label E0 for ϵ < 3.0 eV, E1 for 3.0 eV ≤ ϵ < 3.5 eV, E2 for 3.5 eV ≤ ϵ < 4.0 eV, and E3 for ϵ ≥ 4.0 eV.

**Figure 3:**
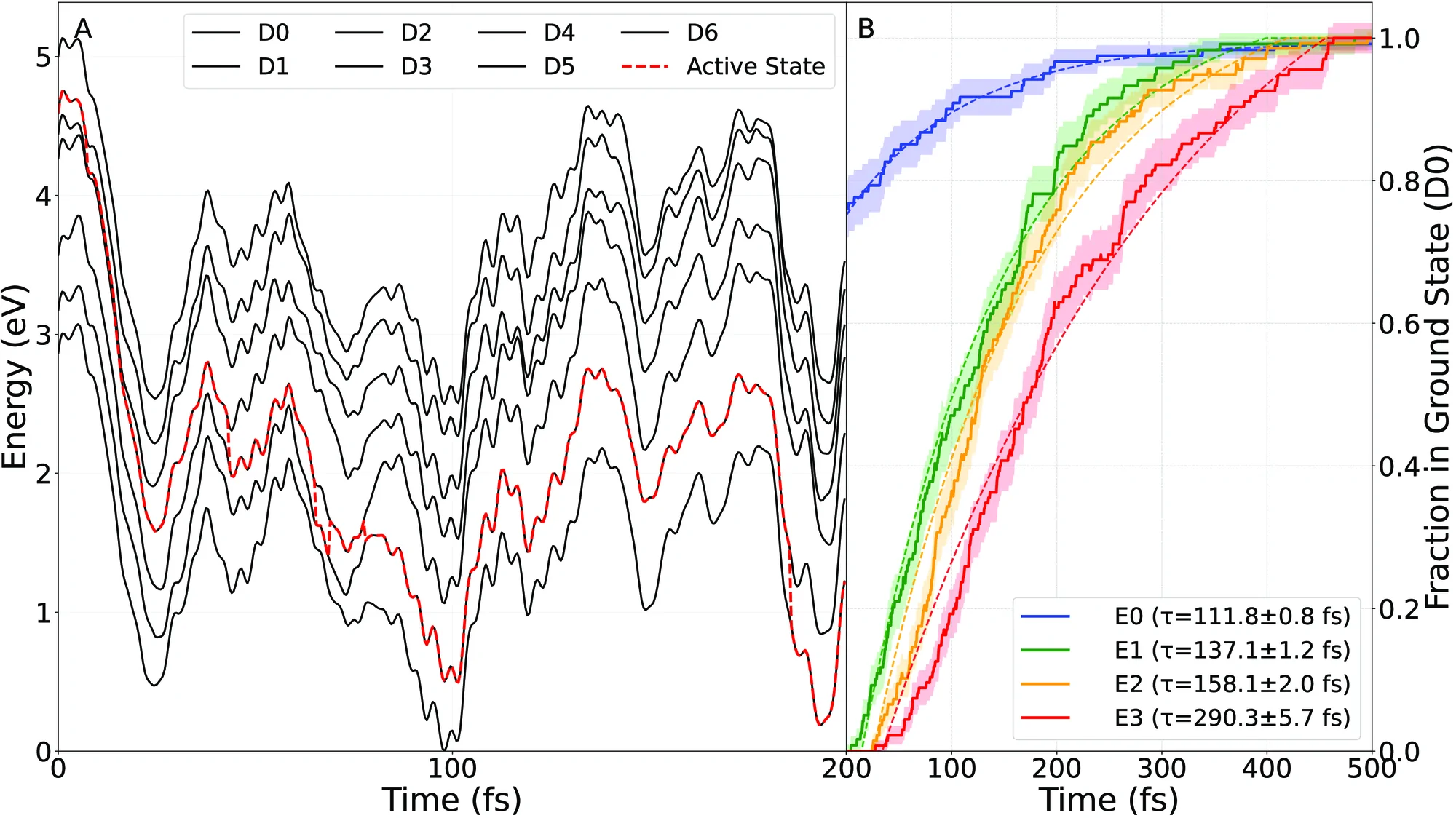
(A) Evolution of the potential energies (electronic states D0-D6) for a typical trajectory with the active state labeled in red. (B) Fraction of trajectories in which the active state is the electronic ground state. Dashed lines indicate exponential fits revealing the time constants given in the legend.

Figure 3(B) shows the population of the ground state as a function of time for ensembles of trajectories classified by the injection energy ϵ. The lowest injection-energy bin E0 represents to a large degree (about 80%) the anionic ground state. For the other injection-energy bins, the electronic relaxation occurs within a few 100 fs. For higher injection energies (E3) it takes longer than for lower injection energies (E1, E2) to reach the electronic ground state. An exponential fit reveals time constants for the electronic relaxation to the ground state of about 137 fs, 158 fs, and 294 fs for E1, E2, and E3 respectively.

Figure 4(A) shows the fraction of trajectories with a nonsolvated electron for the different injection energy bins. One can see that for these energies, the excess electron happens to solvate on time scales of 45.0 fs, 163.8 fs, 205.6 fs, and 392.0 fs, respectively. For all the considered initial conditions and initial injection energies, we find that the electronic state relaxes to the ground state before the excess-electron density contracts. More specifically, we observe that *R*_*G*_ > 3.0 Å as long as the active state is not the electronic ground state. The time evolution of the ground-state fraction in Figure 3 also does not display any structure that would indicate the transient population of a metastable precursor state. Accordingly, we cannot confirm the transient formation of extended double-cavity–*p*-like structures as reported in ref (^22^) due to the limited size of the QM region. Notably, the time scales observed for the electronic relaxation are comparable to the one reported in ref (^22^), but are somewhat larger than the one reported in ref (^29^).

**Figure 4:**
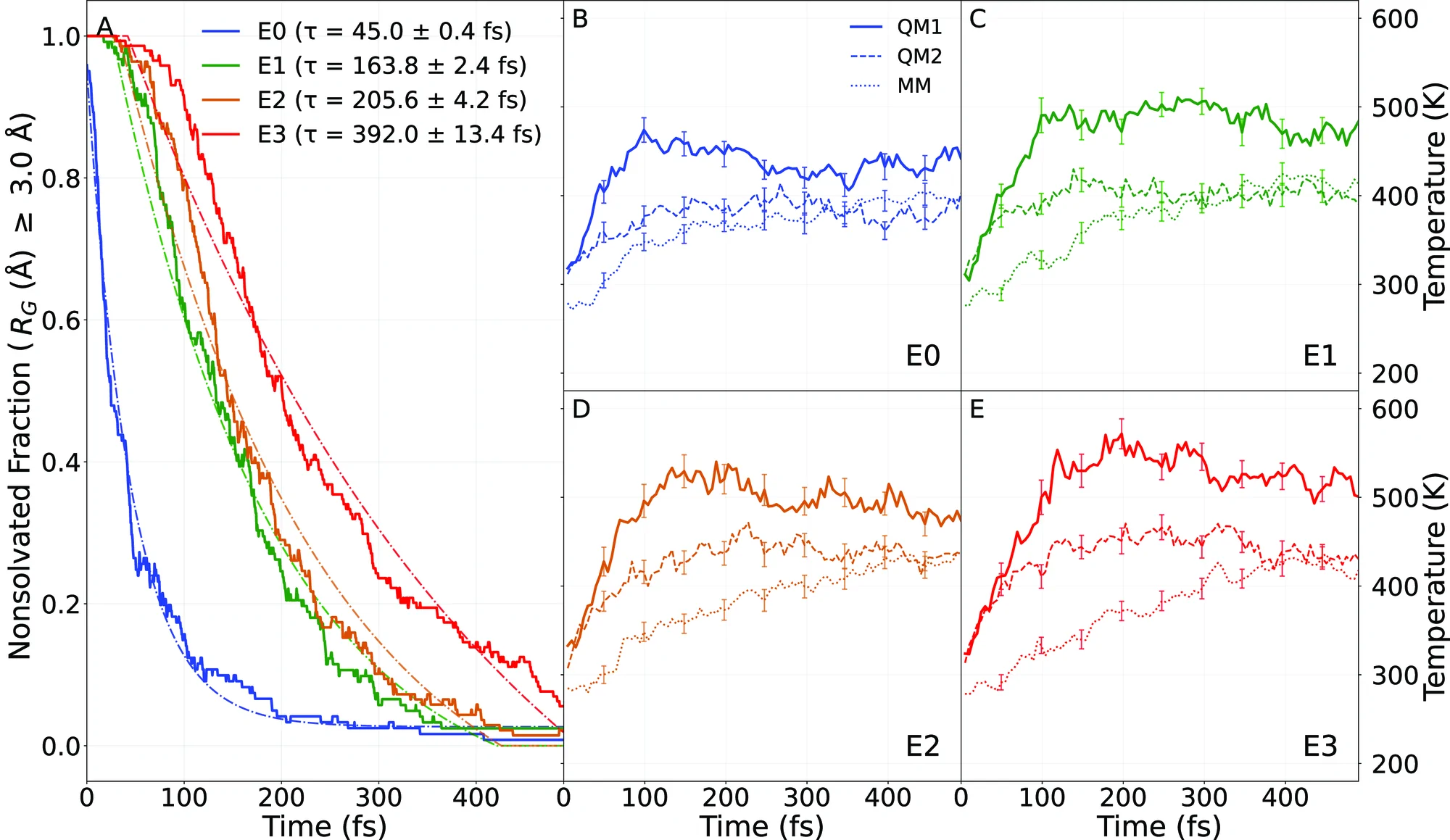
Electron hydration dynamics and local temperature evolution following electron injection. (A) Fraction of nonsolvated electrons as a function of time for different ranges of excitation energy for the injected electron (E0, E1, E2, and E3). Solid lines show the computed fractions, while dashed lines represent monoexponential fits with characteristic time constants (τ) indicated in the legend. (B–E) Local effective temperature evolution in three distinct solvation regions: QM1 (6 closest waters to the center of spin density of the injected electron, solid line), QM2 (remaining 6 QM waters, dashed line), and MM (6 closest MM water molecules, dotted line) for the different injection energy bins. Error bars represent standard errors of the mean.

Our simulations reveal that the solvation dynamics of the excess electron are markedly slower when the electron is initially in a more highly excited state, as evidenced by the delayed contraction of the radius of gyration. At the same time, we observe that the excess electron is initially more delocalized when injected at higher injection energy as compared to when it is injected at the lowest injection-energy class E0. The relation between initial radius of gyration and solvation time follows the same trend as shown in Figure 2 (see Section S2 in the SI), suggesting that the same mechanism is active: Due to its larger degree of delocalization in the excited state, the excess electron’s ability to force nearby water molecules to reorient themselves is lower than in the electronic ground state. In turn, nonadiabatic relaxation to the ground state implies stronger localization of the excess electron, explaining why the time scale for nonadiabatic electronic relaxation determines the time scale for cavity formation and excess-electron density contraction. One can conclude that the need for electronic relaxation substantially delays electron solvation.

The injection of an electron into water implies considerable energy transfer to the nuclear degrees of freedom. The geometric arrangement of the water molecules relaxes on the anionic potential energy surfaces. Furthermore, injection into higher excited states implies additional vibrational and librational energy through internal conversion as illustrated in Figure 3(A). Overall, we find that an energy of about 4 eV is on average transferred to the water within the first 500 fs simulation time. This rapid energy transfer induces local heating, transiently elevating the nuclear kinetic energy of water molecules in the vicinity of the excess electron. In order to assess the amount of local-temperature increase, we computed the instantaneous effective temperature for groups of water molecules based on the kinetic energy of their atoms. Specifically, we consider the group of the six water molecules of the QM region closest to the center of spin density at the time of solvation (QM1), the remaining water molecules of the QM region (QM2), and the 6 MM water molecules closest to the center of spin density. As can be seen in Figure 4(B–E), in the immediate vicinity of the solvated electron (QM1) the temperature reaches values of up to 476 K at 94.5 fs, 500 K at 150 fs, 540 K at 186.7 fs, and 570 K at 176.9 fs for E0, E1, E2, and E3, respectively. The created thermal energy diffuses into the bulk over several hundred femtoseconds, as can be seen by the delayed and somewhat lower temperature rise of the QM2 and MM groups. This mechanism mirrors the ionization-induced heating reported in ref (^44^), where valence ionization in liquid water produced comparable transient thermal gradients. A substantial temperature gradient between the different regions remains after 500 fs, indicating that the water around the solvated electron is still in the process of cooling down.

Our results reveal that the time for the contraction of the excess-electron density is not governed by a single mechanism, but emerges from a critical interplay between its electronic relaxation and the reorganization of the solvent. We identify two competing factors: first, the finite time scale required for the excess electron to relax to its ground state acts as a primary determinant, setting a fundamental lower limit for the solvation process. Second, the energy dissipated during this electronic relaxation creates a pronounced transient thermal environment—evidenced by local temperatures exceeding 500 K—which, in principle, accelerates solvent reorganization by enhancing structural fluctuations and lowering energy barriers.^9^ The eventual time for the excess-electron-density contraction is thus a consequence of this trade-off. The thermal facilitation, while significant, cannot fully compensate for the delay inherent in the prior electronic relaxation step.

In conclusion, our investigation underscores the critical role of the initial quantum delocalization of the excess electron as well as its initial electronic state in dictating its solvation dynamics in liquid water. By varying the basis set within a QM/MM framework, we demonstrate that an initially more localized excess electron leads to faster solvation, consistent with the picture that a locally higher excess-electron density involves a stronger interaction with the nearby water molecules and, thus, more rapidly alters the local water structure.

Our findings further reveal that the solvation dynamics are governed by an interplay between the electronic state and the surrounding water structure. Excited state simulations show that electronic relaxation on sub-300 fs time scales significantly modulates local cavity restructuring and heat dissipation. The results indicate that nonadiabatic transitions are a decisive factor for determining the time scale for electron solvation.

The reported time scale in this work addresses the contraction of the excess-electron density that is typically seen as characteristic for the trap-seeking step in the electron-solvation process. However, it must be said that trap seeking and trap digging cannot be entirely separated, since the geometry of the water molecules and the electronic structure (specifically, the excess-electron density) are adiabatically connected with each other. Accordingly, when the excess electron’s initial degree of delocalization is low, more emphasis is put on an active role of the excess electron. Our result, which shows that faster contraction occurs when the excess electron’s initial radius of gyration is smaller, points to a trap-digging mechanism.

We note that combining these effects together, yields excess-electron-density contraction time scales consistent with the cavity-formation time of 260 fs observed by time-resolved XAS,^9^ assuming that the employed 800 nm-strong-field-laser pulse produces electrons that are not too delocalized and have an injection energy somewhat above the ground state of the conduction band. Such a time scale is also compatible with electron-solvation times inferred from other spectroscopic measurements.^10–18^

## Computational Details

In order to study electron-solvation dynamics, we took water structures sampled from MD calculations similar to those in refs (^44^) and (^45^). To that end, we performed an MD simulation on a 3 nm × 3 nm × 3 nm box containing 884 water molecules using GROMACS^46^ and employing the SPC/E force field.^47,48^ The simulation was based on a leapfrog integrator, and propagated for 2,000 steps with a time step of 5 fs. Holonomic constraints were applied to covalent bonds involving hydrogen atoms,^49^ to ensure computational efficiency and stability. A grid-based neighbor search was used, with a neighbor-list update occurring every 10 steps to address nonbonded interactions in the system. Both short-range van-der-Waals and electrostatic interactions were truncated at 1.0 nm, while long-range electrostatics were handled using the Particle-Mesh-Ewald method^50^ with a Fourier grid spacing of 0.16 nm. The system temperature was maintained at 300 K using a V-rescale thermostat (modified Berendsen thermostat), which efficiently coupled the system to the desired temperature. Isotropic control of pressure was achieved at 1.0 bar using the Parrinello–Rahman barostat.^51^ The compressibility of the system was set to 4.5 × 10^–5^ bar^–1^, which is representative of the physical properties of liquid water. XYZ PBC (3D PBC) were used.

After initial equilibration runs, we continued the simulation based on the QM/MM approach. We divided the simulation box into a QM region and an MM region, selecting 12 spatially connected molecules for the QM region and assigning the remaining water molecules to the MM region. The QM/MM calculations were performed with the ONIOM embedding scheme with the SPC/E force field, where the QM region was treated using the Hartree–Fock method and the 6-31+G^36,37^ basis set. 100 trajectories sampling different QM clusters were performed for 1 ps with a time step of 0.5 fs. In contrast to the preceding SPC/E-based MD simulation, in these QM/MM runs, the internal degrees of freedom (OH bond length and HOH angle) of the QM water molecules were not subject to constraints. The convergence of these internal degrees of freedom is discussed in Section S3 in the SI.

The atomic velocities and positions for different trajectories of the system were then used as initial conditions for the simulation of the electron solvation. With the QM region centered in the simulation box, we continued the simulation without PBC. Water molecules were incorporated into the MM region up to a distance of 10.58 Å from the center of mass of the QM region. We injected the electron by switching to the anionic ground state described by the UHF method employing different basis sets. We ran calculations for 500.0 fs at a time step of 0.5 fs. The total number of trajectories simulated was 90 for the 6-31+G basis set, 58 for the 6-31++G basis set, 40 for the cc-pVDZ basis set, and 56 for the aug-cc-pVDZ basis set.

The relaxation dynamics of the electron starting from electronically excited states were investigated using the FSSH method. Starting from the same setup as prepared for the Born–Oppenheimer trajectories, a number of independent trajectories were propagated for 500 fs with a 0.5 fs time step using the 6-31+G basis set. For the description of the excited state we employed Koopmans’ theorem for electron attachment. Specifically, the energy of the *i*th anionic state is described as

1 $$E_{i}=E_{0}+\epsilon_{i}$$

where *E*_0_ is the energy and ϵ_*i*_ is the *i*th virtual orbital obtained from the neutral Hartree–Fock ground state. The computation of gradients and nonadiabatic coupling vectors for this scheme requires solving the coupled-perturbed Hartree–Fock equations. The FSSH method combined with Koopmans’-*N* + 1 states is implemented in the Chemical Dynamics Toolkit (CDTK) code^52^ linked to the electronic structure code XMOLECULE.^53,54^ At every time step, gradients and nonadiabatic-coupling vectors were computed in order to propagate the wave function and the molecular geometry. For the different initial conditions, we computed trajectories starting at the anionic ground state and the five lowest excited states. Overall, for each of the considered excitation classes (ϵ_*i*_ < 3.0 eV, 3.0 eV ≤ ϵ_*i*_ < 3.5 eV, 3.5 eV ≤ ϵ_*i*_ < 4.0 eV, ϵ_*i*_ ≥ 4.0 eV) more than 120 trajectories were obtained.

In order to study the localization dynamics of the excess electron, we computed the spin density for each trajectory at all time steps and evaluated the center of mass and the radius of gyration *R*_*G*_ as functions of time. Following analysis procedures from prior work,^9,31^ we applied a binary classification scheme to quantify the excess-electron contraction dynamics: at each time point, trajectories were considered to have completed electron solvation, when the radius of gyration of the spin density was below a threshold. The thresholds were set at 3.0 Å for the cc-pVDZ and 6-31+G basis sets and 3.5 Å for the aug-cc-pVDZ and 6-31++G basis sets, reflecting the systematically larger spatial extent of spin density in diffuse basis sets (see Section S1 in the SI for detailed rationale).

## Supplementary Material



## Notes

Analysis and discussion of the radial distribution function of the spin density, additional analysis of the radius of gyration of the spin density for excited-state simulations, analysis of convergence of the OH bond length and HOH bond angle values, analysis of the orientation of water molecules around the excess electron, analysis of the electron localization relative to the QM region, and further results for larger QM regions (PDF)

## #

N.O. is on leave from Department of Physics, Delta State University, Campus III, 330106 Abraka, Nigeria.
