# Supplementary material for: Impact of Initial Electron Localization on Electron Solvation Dynamics in Liquid Water
Source: J Phys Chem Lett. 2026 Jun 26;17(33):9608–15. doi: 10.1021/acs.jpclett.6c01216 (PMC13501955; doi:10.1021/acs.jpclett.6c01216)
Supplement: Supplementary file 1 [file jz6c01216_si_002.pdf]

Supporting Information: Impact of Initial Electron Localization on Electron Solvation Dynamics in Liquid Water

Nathaniel Okpara,[*,†,‡,§] Mathilde Goullieux,[†] Ludger Inhester,[*,†,¶] and Robin Santra[*,†,‡,¶]

†*Center for Free-Electron Laser Science CFEL, Deutsches Elektronen-Synchrotron DESY, Notkestr. 85, 22607 Hamburg, Germany*

‡*Department of Physics, University of Hamburg, Notkestr. 9–11, 22607 Hamburg, Germany*

¶*The Hamburg Centre for Ultrafast Imaging, Luruper Chaussee 149, 22761 Hamburg, Germany*

§*On leave from Department of Physics, Delta State University, Campus III, 330106, Abraka, Nigeria*

* E-mail: nathaniel.okpara@desy.de; ludger.inhester@cfel.de; robin.santra@cfel.de

S1 Radial distribution functions of the spin density

The radial distribution functions (RDFs) of the electron-spin density (Figs. S1 and S2) reveal a universal solvation signature across all four basis sets. Figure S1 shows the RDFs for the 6-31+G [Fig. S1(A)] and the 6-31++G [Fig. S1(B)] basis sets, while Fig. S2 shows the RDFs for the cc-pVDZ [Fig. S2(A)] and the aug-cc-pVDZ [Fig. S2(B)] basis sets for the ensemble of UHF trajectories. In each figure, three different simulation times (top panel: 5 fs, middle panel: 100 fs, bottom panel: 500 fs) are shown with multiple trajectories overlaid. Curves are color-coded based on the radius of gyration of the spin density R_G. Black lines indicate compact electron distributions [$R_G \leq 3.0$ Å for Figs. S1(A) and S1(B); $R_G \leq 3.5$ Å for Figs. S2(A) and S2(B)], whereas red lines represent extended distributions ($R_G >$ threshold for each set).

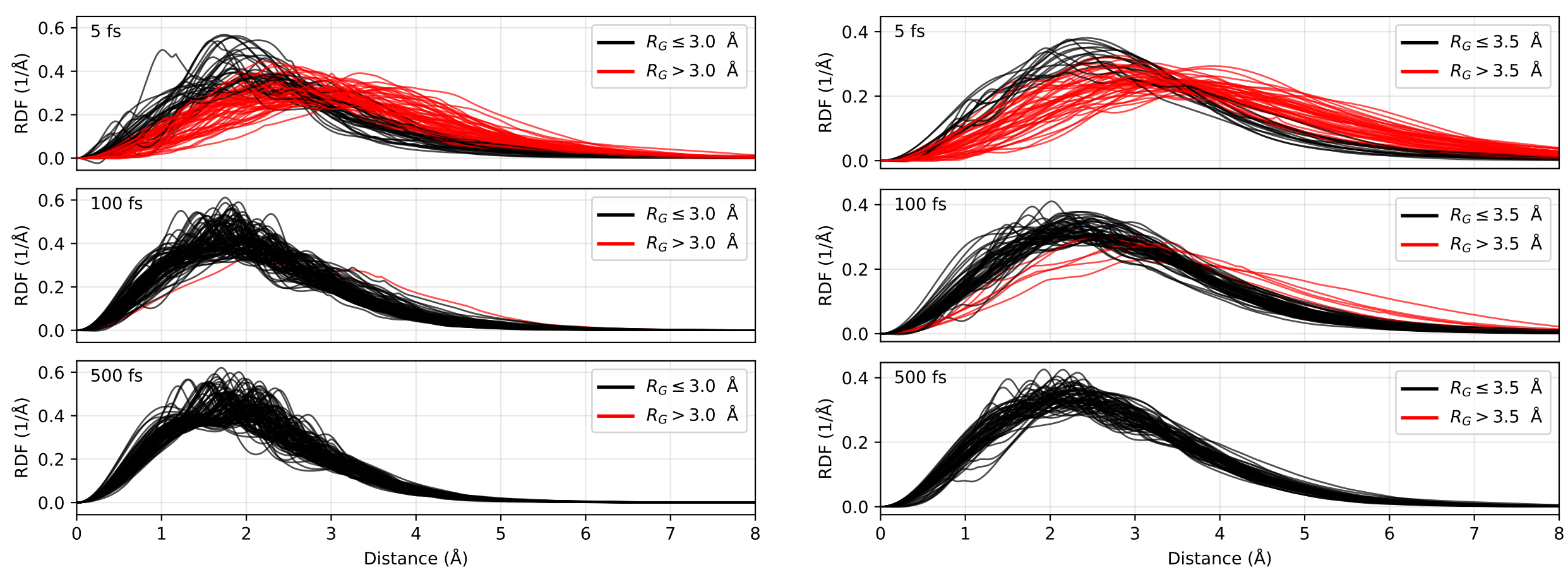

(A) 6-31+G

(B) 6-31++G

Figure S1: Radial distribution functions of electron spin density with R_G-based classification for (A) 6-31+G and (B) 6-31++G at selected times after injection. Black lines: $R_G \leq$ threshold (3.0 Å and 3.5 Å, respectively); red lines: $R_G >$ threshold.

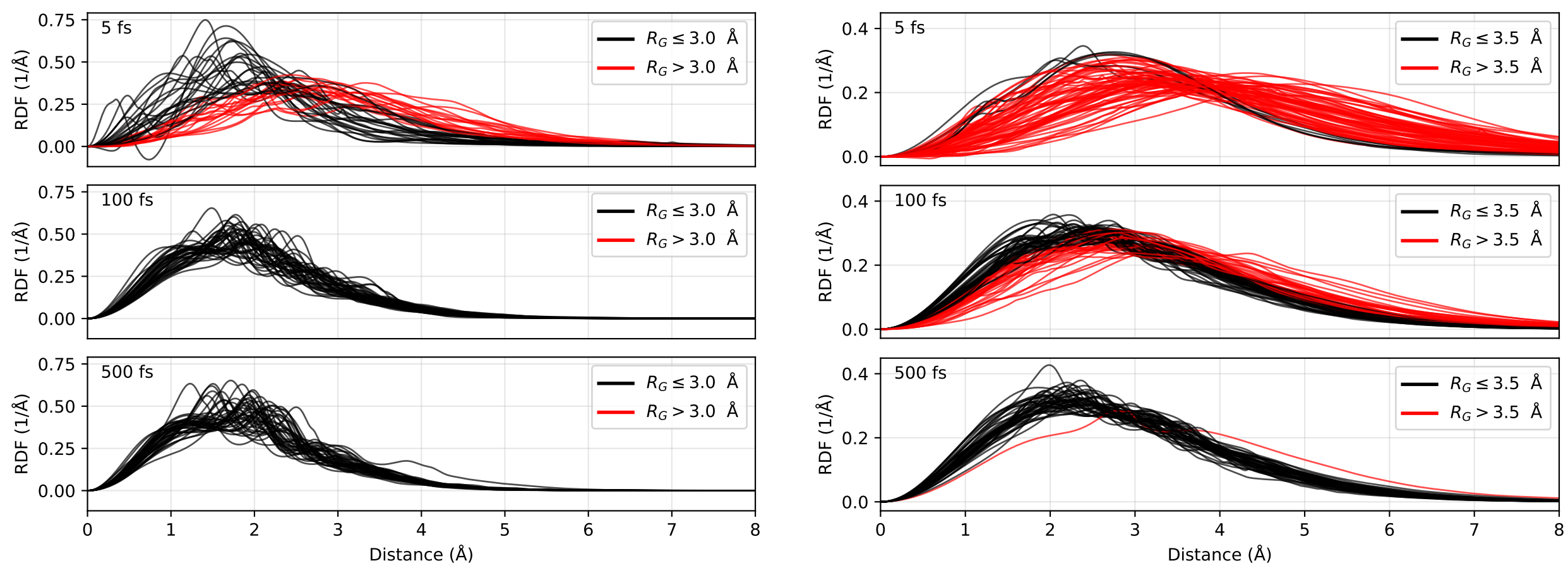

(A) cc-pVDZ (B) aug-cc-pVDZ

Figure S2: Radial distribution functions of electron spin density with R_G-based classification for (A) cc-pVDZ and (B) aug-cc-pVDZ at selected times after injection. Black lines: $R_G \leq$ threshold (3.0 Å and 3.5 Å, respectively); red lines: $R_G >$ threshold.

Each set of plots shows a clear transition from a broad, multi-peaked distribution at 5 fs to a compact, and almost single-peaked profile, signifying a localized, cavity-bound state. The fact that the RDFs remain almost unchanged between 100 fs and 500 fs confirms that a stable solvation state is achieved. This consistent morphological evolution demonstrates that cavity formation is a general physical outcome in our simulations, independent of the specific basis set used.

Despite these universal solvation dynamics, the spatial extent of the localized electron differs systematically. The compact basis sets (6-31+G and cc-pVDZ) yield a more compact spin density than the diffuse basis sets (6-31++G and aug-cc-pVDZ). In particular, for the diffuse basis sets, the solvated electron exhibits an extended tail at distances beyond 4 Å. We note that a similar observation with respect to diffuse functions in the basis set is also reported in Ref. 1 based on minimal anionic water clusters. The R_G metric integrates the entire spatial distribution of the electron density, making it specifically sensitive to the basis-set-dependent differences in the tail at large distances from the center. At the same time, the RDF peak position and the structure at small distances from the center is consistent

across the employed basis sets.

The values for R_G that we observe in Fig. 1 (main text) are generally larger than those reported for minimal water clusters in Ref. 1 and are also larger than values estimated from experiment.[2] Note that the cavity structures considered in Ref. 1 are ideal structures that correspond to a temperature of absolute zero. The fact that our spin density distributions are somewhat more spatially extended can be attributed to the fact that the water structure is still in a relatively hot state; the cavity structure is expected to shrink further as the system cools down.

S2 Radius of gyration of the spin density for excited-state simulations

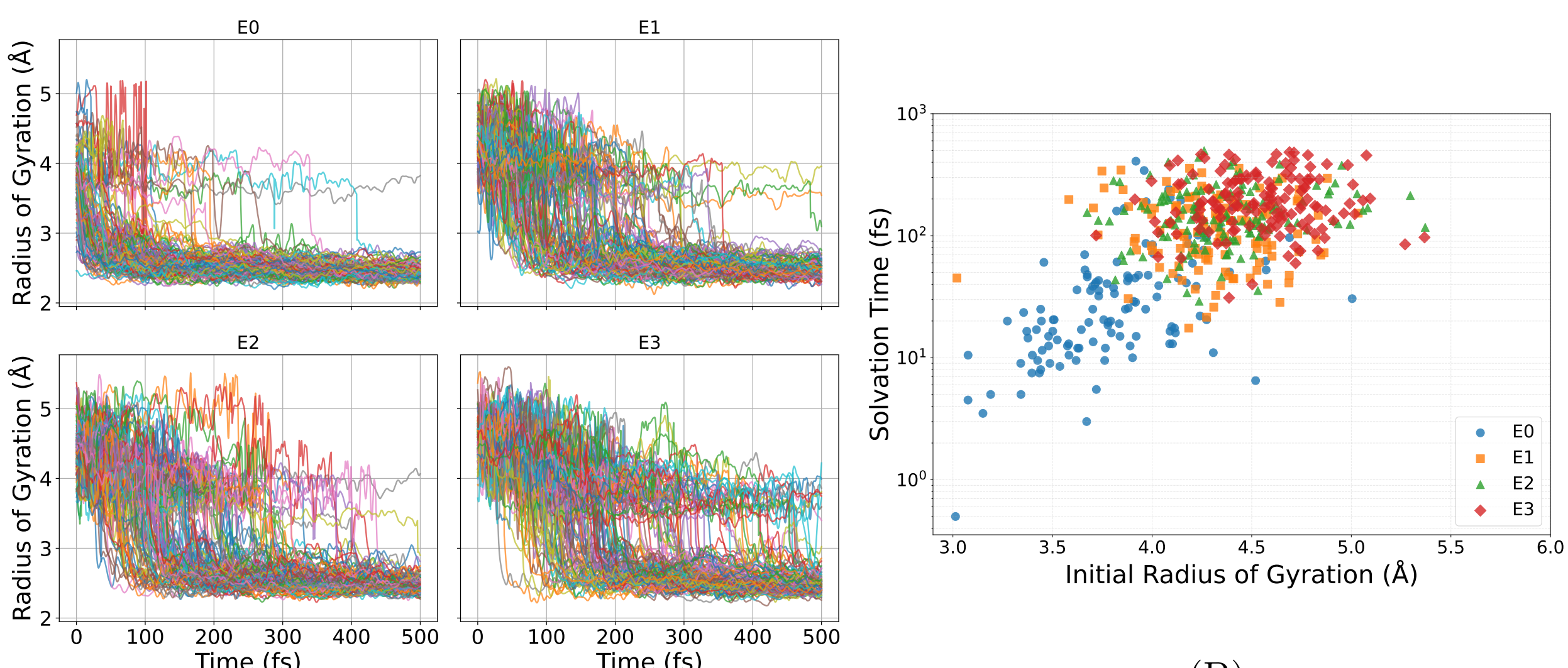

(B)

(A)

Figure S3: (A) Time evolution of the radius of gyration of the spin density and (B) scatter plot showing solvation time vs. initial radius of gyration of the spin density for different ranges of excitation energy for the injected electron (E0, E1, E2, and E3). The solvation time is defined by the time when the radius of gyration drops below a specific threshold (see main text). Note the logarithmic scale used for the vertical axis in panel (B).

S3 Convergence of the OH bond length and HOH bond angle values

In order to discuss the quality of the intermediate QM/MM equilibration, we discuss here the equilibration of the OH bond length and HOH bond angles.

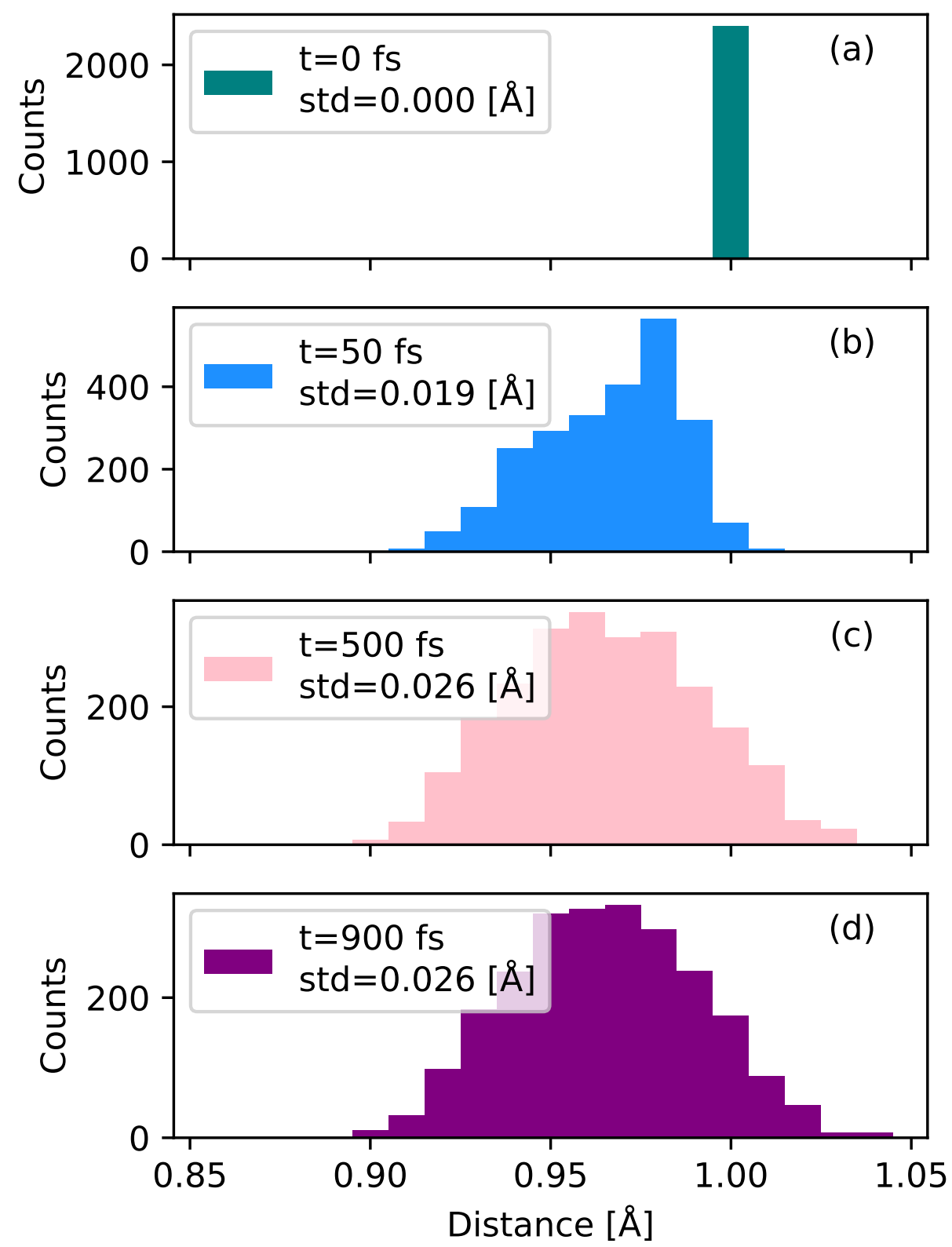

Figure S4: Histogram of OH bond length values at different time steps

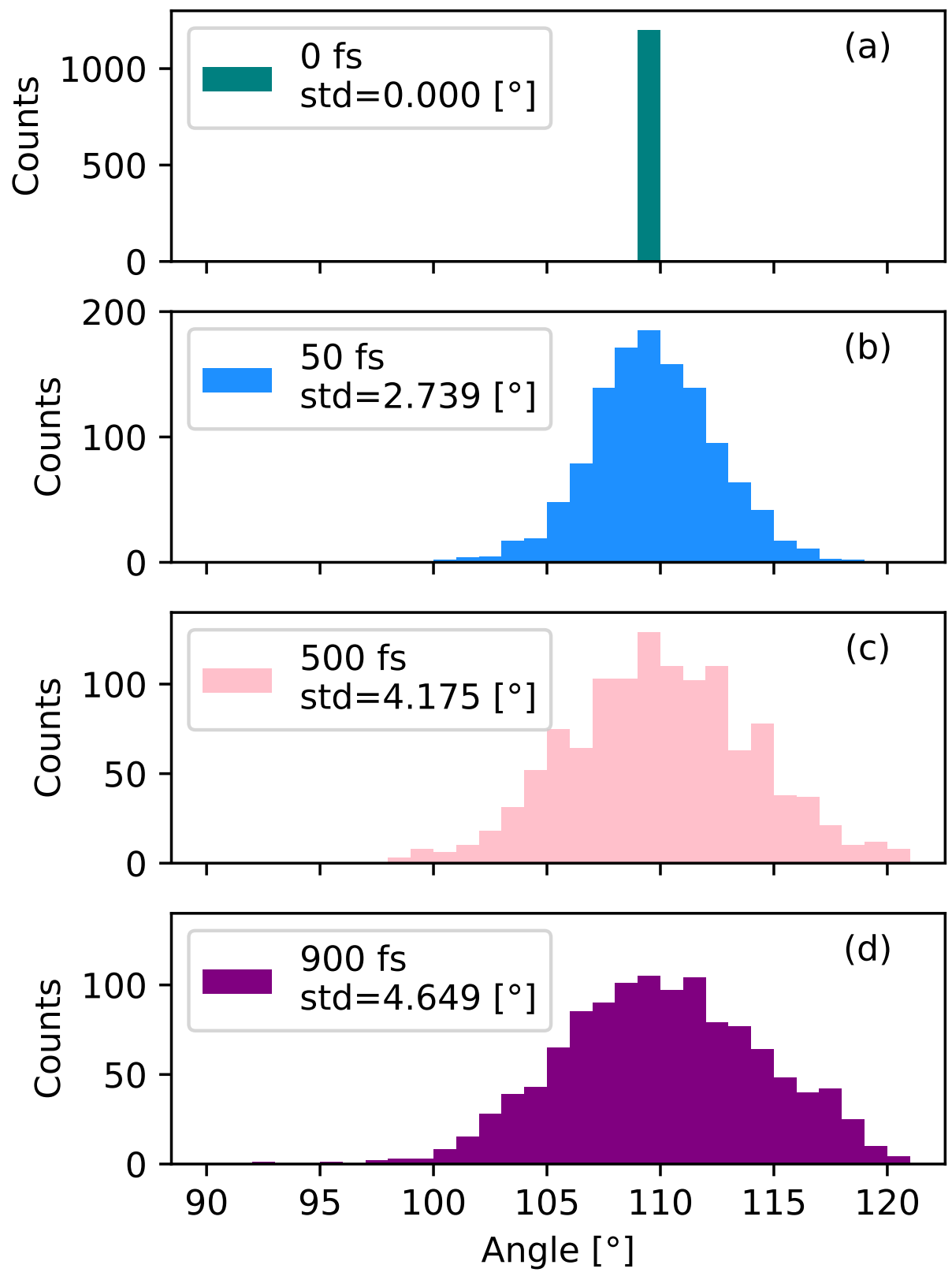

Figure S5: Histogram of HOH angle values at different time steps

Figures S4 and S5 show histograms of the OH bond length and HOH bond angles of the water molecules in the QM region for different snapshots of the 100 QM/MM equilibration runs. At simulation time $t = 0$ fs [Figs. S4(a) and S5(a)], only a single bond length and a single angle are found as determined by the force-field constraints. At $t = 50$ fs [Figs. S4(b) and S5(b)], the distributions already show a considerable spread and, for the bond lengths, a slight shift to lower values. At $t = 500$ fs [Figs. S4(c) and S5(c)], the distributions have slightly changed compared to the situation at $t = 50$fs. In contrast, there is insignificant difference in the distributions between the time steps $t = 500$ fs and $t = 900$ fs [Figs. S4(d) and S5(d)]. This trend is also quantified by the computed standard deviation. We conclude that an equilibration time of 1 ps is sufficient to equilibrate the internal degrees of freedom of the QM water molecules.

S4 Orientation of the water molecules around the excess electron

For the UHF trajectories, we analyzed the orientational order parameter $\cos\theta$ of nearby water molecules of the QM region, where θ is the angle between the water dipole moment and the vector from the oxygen atom to the spin-density center.

Figures S6 and S7 show the time evolution of $\cos\theta(t)$ together with the time evolution of the nonsolvated fraction of trajectories. A value of $\cos\theta \simeq 1$ indicates that water dipoles point with their hydrogens toward the center of the excess electron. We see that $\cos\theta(t)$ starts near 0.2 and rises to a steady-state value around 0.75 within few femtoseconds. This shows that the water molecules reorient themselves in the presence of the excess electron. The observed trend indicates that water molecules in the vicinity of the excess-electron density build up a solvation shell with dipoles preferentially pointing towards the excess-electron. This results is consistent with the emergence of a cavity structure, where 4 - 6 water molecules stabilize the hydrated electron with their OH bonds directed inward. We note that it is not precisely the dipole of the water molecule that is oriented to the center of the excess electron, but rather one of the OH bonds. Nevertheless, the relative orientation of the water dipole reflects the reorientation.

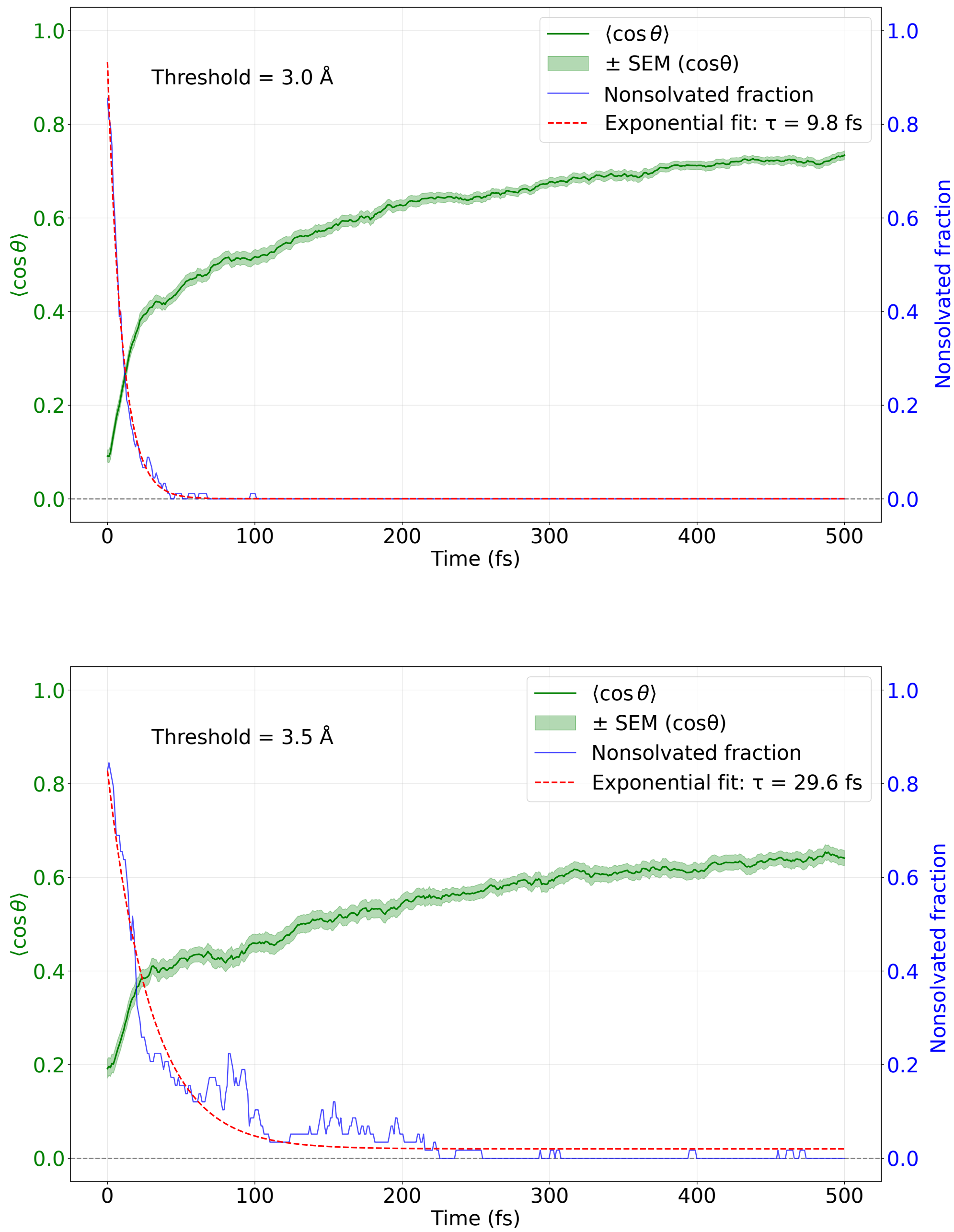

Figure S6: Time evolution of the mean orientation of the 6 nearest water molecules in the QM region and the fraction of nonsolvated cavities for the 6-31+G (a) and 6-31++G basis sets (b). Left axis (green): Ensemble-averaged $\cos\theta$. The shaded region represents the standard error of the mean (SEM). Right axis (blue): Fraction of trajectories (same as in Fig. 1 of the main text), in which the radius of gyration of the spin density is below a given threshold. The red dashed lines show exponential fits of the decay of the non-solvated fraction, yielding a characteristic solvation time τ.

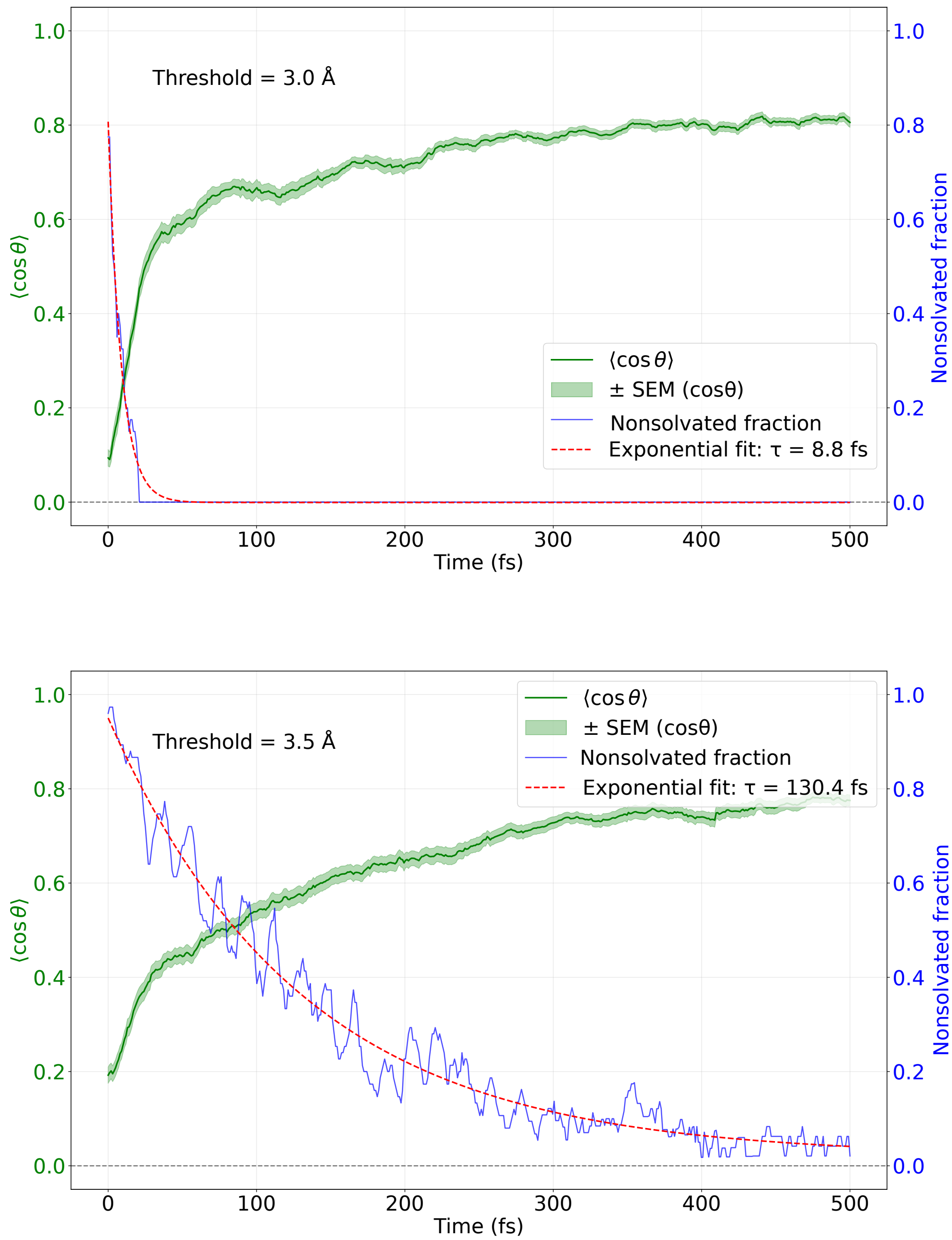

Figure S7: Time evolution of the mean orientation of the 6 nearest water molecules in the QM region and the fraction of nonsolvated cavities for the cc-pVDZ (a) and aug-cc-pVDZ basis sets (b). Left axis (green): Ensemble-averaged $\cos\theta$. The shaded region represents the standard error of the mean (SEM). Right axis (blue): Fraction of trajectories (same as in Fig. 1 of the main text), in which the radius of gyration of the spin density is below a given threshold. The red dashed lines show exponential fits of the decay of the non-solvated fraction, yielding a characteristic solvation time τ.

For all basis sets, we observe that the drop of R_G is accompanied by an increase of $\cos\theta$. Two steps in the evolution of $\langle\cos\theta\rangle$ can be recognized, a rapid increase in the first 20 fs, followed by a somewhat slower increase.

As indicated by the evolution of the nonsolvated fraction, the drop of R_G, i.e., the contraction of the excess-electron density, goes hand in hand with the reorientation of close-by water molecules. This illustrates that the dynamics of the spin density are directly coupled with the first steps of cavity formation. After the radius of gyration of the excess electron has collapsed, the solvation dynamics continue and further water molecules adapt their orientation with respect to the center of the excess electron.

S5 Electron localization relative to the QM region

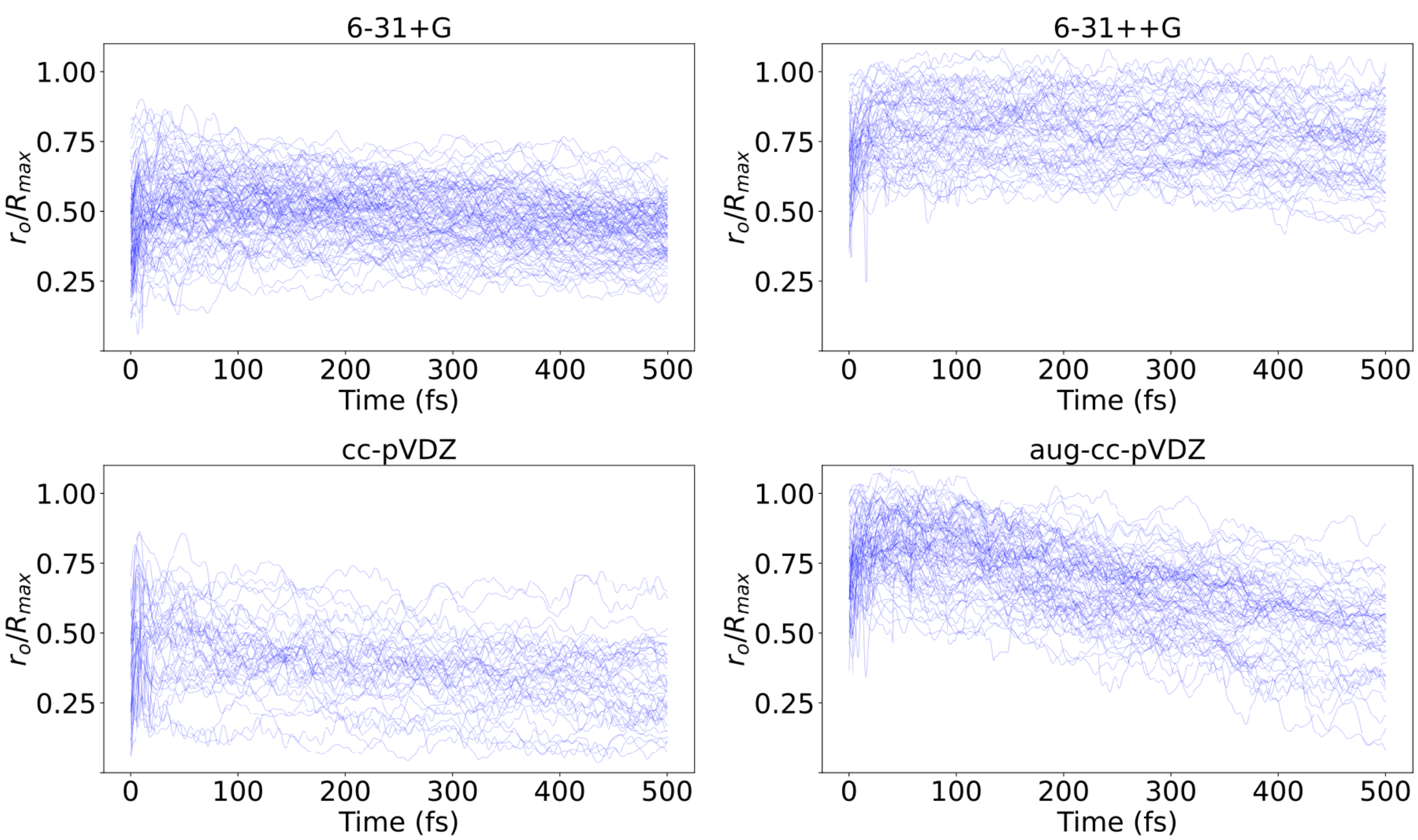

Figure S8: Time evolution of the distance from the excess-electron spin center to the center of mass (COM) of the QM region relative to the maximum distance from the COM to any atom of the QM region.

To investigate the position at which the excess electron contracts during the solvation dynamics, we analyzed the UHF trajectories and computed the distance $r_o(t)$ of the excess-electron center relative to the center of mass of the QM region. This quantity is plotted relative to the maximum distance $R_{max}(t)$ of any atom in the QM region to the center of mass of the QM region .

As can be seen, for the basis sets without additional diffuse basis functions, the ratio $r_o(t)/R_{max}(t)$ has values ranging from 0.2 to 0.8, showing no substantial drift along the simulation time. For the 6-31++G and aug-cc-pVDZ basis sets, the ratio $r_o(t)/R_{max}(t)$ is larger, covering values from 0.5 to 1.0. For the aug-cc-pVDZ basis sets, we see a clear drift of $r_o(t)/R_{max}(t)$ toward lower values.

S6 Further results for larger QM regions

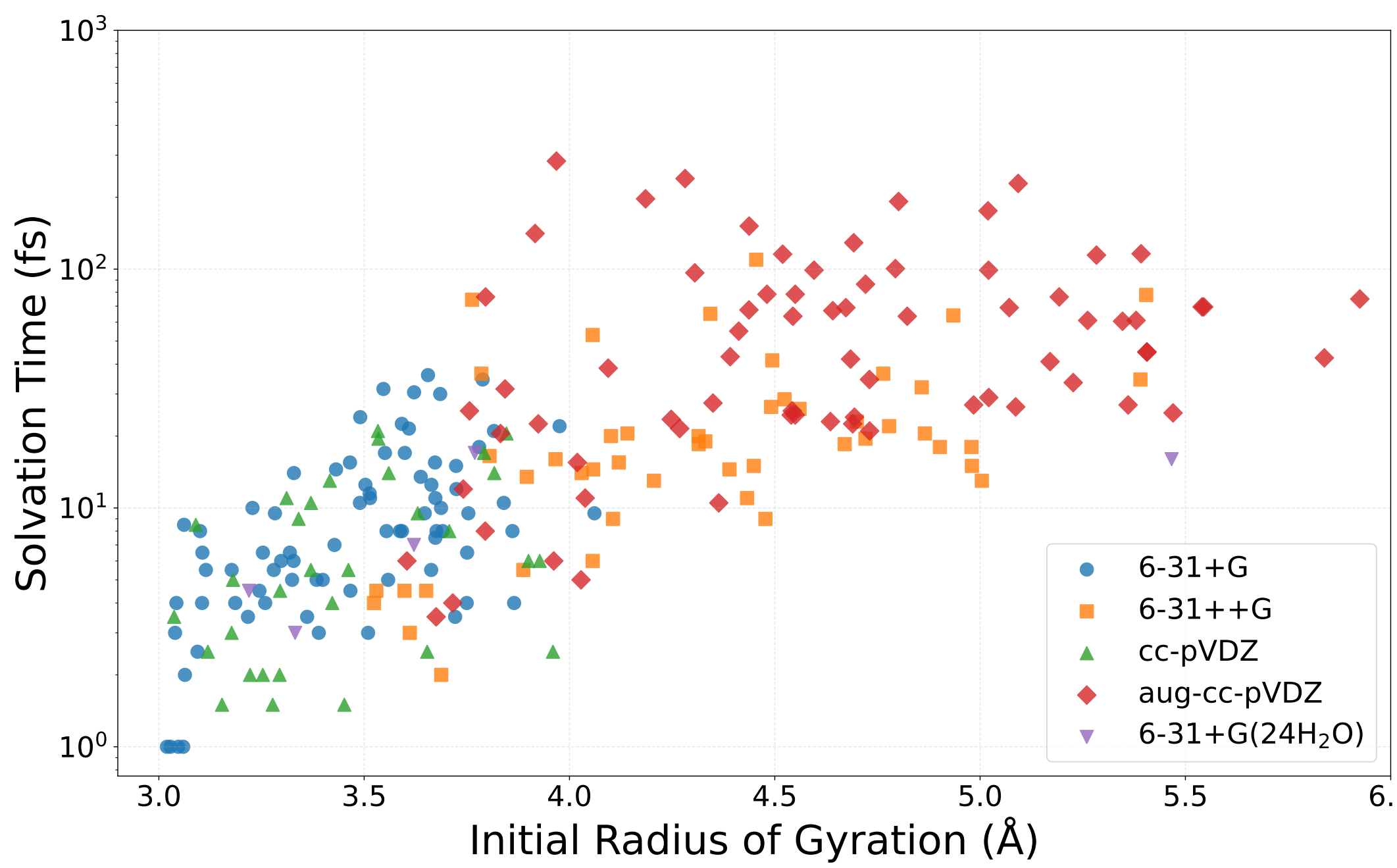

Figure S9: Scatter plot showing solvation time vs. initial radius of gyration of the spin density for each trajectory, as in Fig. 2 in the main text.

Figure S9 shows the same plot as in Fig. 2 of the main text, but incorporating additional data that was extracted from 5 trajectories performed for the 6-31+G basis set and a larger QM region, covering 24 water molecules. For these additional trajectories, the initial radii of gyration of the spin density and the obtained solvation time scales (time at which R_G drops below the threshold) follow a similar trend as for the data obtained with a QM region of 12 water molecules.

References

(1) Kumar, A.; Walker, J. A.; Bartels, D. M.; Sevilla, M. D. A simple ab initio model for the hydrated electron that matches experiment. *The Journal of Physical Chemistry A* **2015**, *119*, 9148–9159.

(2) Bartels, D. M. Moment Analysis of Hydrated Electron Cluster Spectra: Surface or Internal States? *The Journal of Chemical Physics* **2001**, *115*, 4404–4405.
